# Characteristics of gray matter alterations in never-treated and treated chronic schizophrenia patients

**DOI:** 10.1038/s41398-020-0828-4

**Published:** 2020-05-12

**Authors:** Nian Liu, Yuan Xiao, Wenjing Zhang, Biqiu Tang, Jiaxin Zeng, Na Hu, Shah Chandan, Qiyong Gong, Su Lui

**Affiliations:** 1 Huaxi MR Research Center (HMRRC), Functional and molecular imaging Key Laboratory of Sichuan Province, Department of Radiology, West China Hospital, Sichuan University, Chengdu, 610041 China; 2 Department of Radiology, Affiliated Hospital of North Sichuan Medical College, Nanchong, 637000 China

**Keywords:** Clinical pharmacology, Schizophrenia

## Abstract

Though gray matter deficits have been consistently revealed in chronic treated schizophrenia, it is still not clear whether there are different brain alterations between chronic never treated and treated patients. To explore the different patterns of gray matter alterations among chronic never treated patients and those treated with monotherapy, we recruited 35 never-treated chronic schizophrenia patients with illness durations ranging from 5 to 48 years, 20 illness duration-matched risperidone monotherapy and 20 clozapine monotherapy patients, and 55 healthy controls. GM (surface area, cortical thickness, and cortical volume) measures were extracted and compared using ANCOVA across the four groups followed by post hoc tests. Relative to controls, both treated and never-treated chronic schizophrenia patients showed reduced GM mainly involving the bilateral medial and rostral middle frontal, left banks superior temporal sulcus, left fusiform, and left pericalcarine cortex and increased in the left cuneus. Compared with the untreated patient group, the two treated groups showed reductions mainly in the bilateral prefrontal, temporal, and left inferior parietal lobules. The clozapine monotherapy patients demonstrated more severe decreases in the bilateral prefrontal cortex and left cuneus and less severe decreases in the left ventral temporal lobe than risperidone monotherapy patients. These findings provide new insights into the long-term effects of antipsychotic treatment on gray matter alterations in schizophrenia patients. Furthermore, the characteristic findings of reductions in the inferior parietal lobule might be specific for long-term antipsychotic treatment, which could be a possible target for medication development in the future.

## Introduction

Recent advances in psychoradiology^1^ have led to an increase in brain studies of psychiatric disorders, especially in first-episode and chronic schizophrenia patients^2–6^. Previous structural MRI studies^7–9^ have demonstrated that first-episode drug-naive schizophrenia patients showed GM reductions in the prefrontal and temporal lobes, and chronic schizophrenia patients have abnormalities in the frontal, temporal, superior parietal, and occipital lobes^10–15^. Although the above studies have reported inconsistent findings in patients with chronic and first-episode schizophrenia, it is still unclear whether the findings are due to neuroprogressive mechanisms or stage-specific characteristics.

Previous studies have shown GM abnormalities in chronic schizophrenia^10–15^, but these studies have mostly been during the middle course of the illness. However, only two studies^16,17^ reported GM alterations during a relatively later course of the illness. A cross-sectional brain structural study^16^ found that, relative to healthy controls, schizophrenia patients with an untreated illness course of more than 20 years demonstrated less cortical thickness in the bilateral ventromedial prefrontal cortices, left superior temporal gyrus, and right pars triangularis and greater cortical thickness in the left superior parietal lobe. However, there has been a lack of direct comparisons between long-term treated and untreated patients, which is crucial for understanding the neurobiological consequences of long-term treatment.

In fact, the short-term effects of antipsychotic treatment have been well studied by a number of longitudinal MRI studies^7,18–20^ and have found progressive GM loss in the frontal, temporal and occipital lobes after 2–3 years of treatment. However, only a few studies have explored longer duration treatment effects, but the findings have been inconsistent^21,22^. Beng-Choon Ho et al.^21^ reported that parietal lobe reductions in schizophrenia were associated with higher doses of non-clozapine atypical antipsychotics in patients over an average follow-up of 7.2 years. However, this finding was not replicated by Veijola et al.^22^. These inconsistent findings may have resulted from different types/doses of antipsychotics. Furthermore, it is still not clear whether there are different characteristic changes in brain structure after long-term antipsychotic medication use in schizophrenia patients. Therefore, the study of monotherapy may shed more light on the effects of long-term treatment on brain structure in schizophrenia patients.

Therefore, to explore the long-term effects of antipsychotic treatment on brain morphology, we recruited 35 never-treated chronic schizophrenia patients with a mean illness duration of 20 years, 20 illness-duration-matched risperidone monotherapy patients, 20 illness-duration-matched clozapine monotherapy patients, and 55 healthy controls. We hypothesize that characteristic or specific GM change patterns would be found both in schizophrenia with risperidone monotherapy and clozapine monotherapy relative to never-treated patients and that brain structures differentially respond to different kinds of antipsychotics.

## Materials and methods

### Participants

Thirty-five never-treated chronic schizophrenia patients with long-term illness (ranging from 5 to 48 years), 20 illness-duration-matched risperidone monotherapy patients, 20 illness-duration-matched clozapine monotherapy patients and 55 healthy controls (HC) were included and assessed using a single GE MR scanner. The diagnosis of schizophrenia was determined with the consensus of two psychiatrists using the Structured Interview for the DSM-IV (SCID). Illness onset was evaluated by the Nottingham Onset Schedule^23^ with the information provided by the patients, other family members, and other sources. Patients with a duration of illness longer than 5 years were defined as “chronic”^24,25^. Symptom severity assessment was carried out using the Positive and Negative Syndrome Scale (PANSS)^26^ on the same day as MRI scanning. This study was approved by the Institutional Review Board, and written informed consent was obtained from each participant.

Never-treated chronic schizophrenia patients were identified from the community Mental Health Screening Program, aiming to identify and provide psychiatric care to individuals with serious but untreated mental illness. Most of these never-treated patients are over 40 years old and were typically recruited from rural or suburban areas within 35 km of downtown Chengdu and Zigong city in Western China. Based on available retrospective information, 9 of these patients had developed acute psychosis after significant life trauma, such as the loss of a close relative or parental marital difficulties. The other 26 patients had a gradual and insidious onset of symptoms. Twenty-one had no involvement in any employment. Fourteen were able to care for themselves in many activities of daily living. Most of the never-treated patients had 6 years of primary education, while the antipsychotic-treated patients had 9 years of education in China. These never-treated patients had not previously received any antipsychotic drug treatment for a variety of reasons, but all of the patients had been well taken care of by their families since the onset of their illness.

The treated patients were recruited from the same or nearby communities and had received antipsychotic treatment relatively consistently beginning early in the course of their illness. The patients had been treated with risperidone or clozapine, and no patient had switched to another antipsychotic in the previous 5 years. The dose of antipsychotic medication was converted to chlorpromazine (CPZ) equivalents^23,27^.

Healthy control subjects (HC, *n* = 55) were recruited to match the patient groups from poster advertisements in the local area of patients, and they had similar socioeconomic and educational backgrounds. All controls were screened using the SCID-Non-Patient Version to confirm the lifetime absence of psychiatric and neurological illnesses, and all participants were right-handed.

The exclusion criteria for patients and healthy controls were as follows: (1) contraindication for MRI examination, (2) another axis I psychiatric disorder, (3) any neurological disorder, (4) lifetime drug or alcohol abuse or dependency, and (5) pregnancy or significant systemic illness, such as hepatitis or cardiovascular disease. MR images of all participants were inspected by an experienced neuroradiologist to exclude subjects with visible brain abnormalities.

### Data acquisition

All MRI data were acquired on a 3.0 T GE Signa EXCITE Scanner (General Electric Medical Systems, Milwaukee, Wisconsin) with an 8-channel phase array head coil. All participants went through a uniform protocol that comprised an axial high-resolution T1-weighted image with a volumetric three-dimensional spoiled gradient-echo sequence with the following parameters: repetition time = 8.5 ms, echo time = 3.4 ms, flip angle = 12^o^, voxel size = 0.47 × 0.47 × 1 mm^3^, the field of view = 240 × 240 mm^2^, matrix = 256 × 256, 156 axial slices, and slice thickness = 1 mm. All participants were told to lie still and keep their eyes closed without thinking anything during scanning. Images were immediately checked by a neuroradiologist, and participants with substantial motion or other artifacts were subjected to reimaging.

### Imaging processing

The FreeSurfer (version 6.0, https://surfer.nmr.mgh.harvard.edu/) package was used to perform cortical modeling and volumetric segmentation and to measure cortical volume and subcortical volume by using image intensities and continuity information from the entire MR volume to construct representations of the gray/white matter boundary and pial surface^28–30^. The processing steps, including skull stripping, segmentation of the gray matter, white matter and CSF, cortical surface reconstruction, registration, and parcellation, were initialized with common information from the within-subject template^29–31^. The above procedure generated average cortical volumes for 68 regions and the subcortical volumes for 14 regions of interest (nucleus accumbens, amygdala, caudate, hippocampus, pallidum, putamen, and thalamus in each hemisphere) using the Desikan–Killiany atlas template^32^. Postprocessing visual inspection for the quality of both imaging processes was reviewed without knowledge of subject characteristics. Cortical thickness and surface area were also investigated, using similar methods.

### Statistical analysis

All statistical analyses were performed using SPSS (version 22.0, U.S.A.). Demographic variables (e.g., age, sex, education) and clinical characteristics (i.e., medications and PANSS scores) were compared among the groups via one-way analysis of variance (ANOVA), two-sample *t*-tests or chi-square tests. For brain imaging analyses, average cortical surface area, cortical thickness, and cortical volume across the 68 regions of interest (ROIs) and the subcortical volumes of 14 regions using the Desikan–Killiany atlas template were extracted. Analysis of covariance (ANCOVA) was conducted using a general linear model (GLM) followed by the least significant difference (LSD) post hoc pairwise comparisons after controlling for age and sex. The statistical significance level was set at *p* = 0.05 with two-tailed, controlled for multiple comparisons using a false discovery rate (FDR) criterion for each of the 68 cortical measurements and 14 subcortical volumes. Pearson correlation analyses among the anatomical measures and drug dose, PANSS scores, and illness duration were performed.

## Results

### Demographic and clinical characteristics

The demographic and clinical characteristics of the study participants are listed in Table 1. There was no significant difference in age and educational years among all the groups. There was also no difference in the duration of illness and age at onset among the never-treated schizophrenia (NT-SCZ) group, risperidone-treated schizophrenia (RT-SCZ) group and clozapine-treated schizophrenia (CT-SCZ) group (all *p* > 0.05). The sex ratio was significantly different among the groups (*p* < 0.01), with the RT-SCZ group having more males than the other groups. Both treated patient groups showed symptom remission relative to the NT-SCZ group (all *p* < 0.01). No significant difference in symptoms was found between the RT-SCZ group and the CT-SCZ group or in the daily dosage of antipsychotic medications based on CPZ equivalents (all *p* > 0.05).

**Table 1:** Table 1. Demographical characteristics of the participants.

Demographic/clinical characters	NT-SCZ (Mean ± SD), *n* = 35)	RT-SCZ (Mean ± SD), *n* = 20	CT-SCZ (Mean ± SD), *n* = 20	HC (Mean ± SD), *n* = 55	ANOVA, *t* or *χ*^2^ *P*-value	Post hoc analysis					
						NT-SCZ vs. HC	NT-SCZ vs. RT-SCZ	NT-SCZ vs. CT-SCZ	RT-SCZ vs. CT-SCZ	RT-SCZ vs. HC	CT-SCZ vs. HC
Male/female	17/18	17/3	10/10	29/26	<0.001	0.198	<0.001	0.880	0.002	<0.001	0.432
Age, years	47.97 (13.41)	45.20 (7.48)	50.05 (4.98)	47.49 (10.33)	0.523	NA	NA	NA	NA	NA	NA
Education, years	7.77 (3.27)	9.60 (2.56)	8.60 (3.35)	9.40 (2.77)	0.053	NA	NA	NA	NA	NA	NA
Duration of illness (months)	242.86 (148.87)	202.08 (99.63)	236.75 (105.71)	NA	0.515	NA	NA	NA	NA	NA	NA
Age at onset, years	27.69 (7.58)	28.30 (8.97)	30.32 (7.39)	NA	0.492	NA	NA	NA	NA	NA	NA
PANSS total score	92.76 (17.43)	50.40 (12.11)	56.95 (11.41)	NA	<0.001	NA	<0.001	<0.001	0.162	NA	NA
PANSS positive symptom	24.76 (6.97)	9.60 (2.64)	10.65 (3.41)	NA	<0.001	NA	<0.001	<0.001	0.529	NA	NA
PANSS negative symptom	24.32 (8.20)	15.75 (5.22)	19.00 (5.08)	NA	<0.001	NA	<0.001	0.007	0.132	NA	NA
General psychopathology symptom	43.68 (7.94)	25.05 (5.17)	27.30 (5.21)	NA	<0.001	NA	<0.001	<0.001	0.285	NA	NA
Antipsychotic dosage (CPZ equivalent, mg/day)^a^	NA	427.50 (131.26)	357.19 (161.81)	NA	0.140	NA	NA	NA	NA	NA	NA

Statistical tests performed: group differences in age, education, duration, age at onset and PANSS score, One way ANOVA test and post hoc test; gender, chi-square test; antipsychotic dosage, independent sample *t*-test. *PANSS* positive and negative syndrome scale, *CPZ* chlorpromazine, *HC* healthy controls, *NT-SCZ* never treated schizophrenia patients, *RT-SCZ* risperidone-treated schizophrenia patients, *CT-SCZ* clozapine-treated schizophrenia patients.

^a^ Daily dosage of antipsychotic medications in chlorpromazine equivalent during the last 4 weeks of treatment^27^.

### ANCOVA findings among four groups

GM measurements (cortical volume, surface area, or thickness) demonstrated a significant main effect among the four groups by analysis of covariance (ANCOVA) after multiple comparisons, involving 46 brain regions, but excluding the bilateral caudal middle frontal, lateral occipital, insula, cingulate, motor and sensory cortex (all corrected *p* < 0.05; see details in Supplementary Table S1), and the variance is similar by the test of homogeneity of variances. There was no significant difference in subcortical volume after multiple comparisons (all corrected *p* > 0.05). The GM characteristics in cortical volumes, thicknesses, and surface areas of all regions are listed in the supplementary materials (Supplementary Table S1).

### Comparison of patients with chronic schizophrenia and healthy controls

**Fig. 1:**
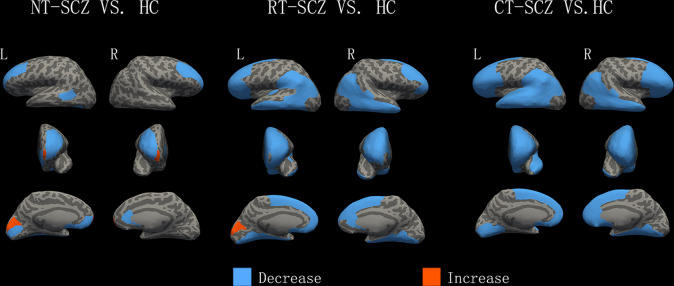
Differences in GM between patients with chronic schizophrenia and healthy controls. Greater cortical volume, surface area, or thickness in patients than in comparison subjects are indicated by red/warm color, and lower volume, surface area, or thickness are indicated by blue/cold color. L, left hemisphere; R, right hemisphere; HC, healthy controls; NT-SCZ, never treated schizophrenia patients; RT-SCZ, risperidone-treated schizophrenia patients; CT-SCZ, clozapine-treated schizophrenia patients.

The post hoc analyses revealed that relative to the HC group, both treated and never-treated chronic schizophrenia patients showed reduced GM mainly involving the bilateral rostral middle frontal and medial frontal cortex, left banks superior temporal sulcus, left fusiform, left pericalcarine regions and increased GM in the left cuneus. The never-treated group showed an additional increase in the bilateral frontal pole. The treated groups showed an additional reduction of GM involving bilateral prefrontal, temporal, parietal lobe, and bilateral lingual cortex (*p* < 0.05, corrected; Fig. 1).

### Comparison of never-treated and treated chronic patient groups

**Fig. 2:**
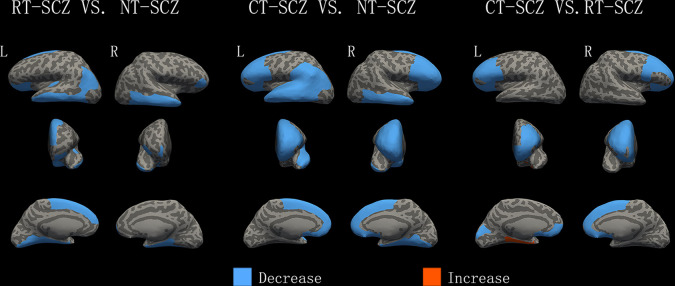
Differences in GM between treated and never treated patients with chronic schizophrenia. Greater cortical volume, surface area, or thickness in treated patients than in comparison subjects are indicated by red/warm color, and lower volume, surface area, or thickness are indicated by blue/cold color. L, left hemisphere; R, right hemisphere; NT-SCZ, never treated schizophrenia patients; RT-SCZ, risperidone-treated schizophrenia patients; CT-SCZ, clozapine-treated schizophrenia patients.

The post hoc analyses revealed that compared with the never-treated patient group, both treated chronic patient groups showed reductions mainly in the left lateral orbitofrontal and superior frontal, right frontal pole, right pars orbitalis, bilateral inferior and middle temporal, right medial temporal lobe, and left inferior parietal cortex regions (*p* < 0.05, corrected; Fig. 2). The RT-SCZ group showed an additional reduction of the GM in the left medial temporal lobe and left lingual cortex, and the CT-SCZ group showed an additional reduction in the bilateral prefrontal lobe, left superior temporal, left supramarginal, and right lingual cortex regions (*p* < 0.05, corrected; Fig. 2).

### Comparison of risperidone and clozapine monotherapy patient groups

The post hoc analyses revealed that the CT-SCZ group showed more decreased GM than the RT-SCZ group in bilateral lateral and medial orbitofrontal, bilateral rostral middle frontal, bilateral pars opercularis, right superior frontal cortex, left pars triangularis, left frontal pole, and the left cuneus cortex regions but less decreased GM than the RT-SCZ group in the left entorhinal and left parahippocampal cortex (*p* < 0.05, corrected; Fig. 2).

### Correlations between alterations in GM in relation to drug dosage, illness duration and symptoms

In the RT-SCZ group, the illness duration was negatively correlated with the left inferior temporal thickness (*r* = −0.533, *p* = 0.015), left inferior temporal volume (*r* = −0.576, *p* = 0.008), left middle temporal volume (*r* = −0.496, *p* = 0.026), and left inferior parietal volume (*r* = −0.531, *p* = 0.016) (Table 2). The left middle temporal thickness was related to the dosage (*r* = −0.500, *p* = 0.025), PANSS total scores (*r* = −0.487, *p* = 0.029), negative syndrome scores (*r* = −0.473, *p* = 0.035), and general psychopathology scores (*r* = −0.502, *p* = 0.024). The left parahippocampal thickness was related to positive syndrome scores (*r* = −0.479, *p* = 0.033). In the CT-SCZ group, the illness duration was negatively correlated with the right fusiform thickness (*r* = −0.486, *p* = 0.030) and right inferior temporal volume (*r* = −0.607, *p* = 0.005). The left frontal pole thickness was related to the PANSS total scores (*r* = −0.523, *p* = 0.018) and negative syndrome scores (*r* = −0.622, *p* = 0.003). The alterations in GM were not associated with illness duration or clinical symptoms in the NT-SCZ group (*p* > 0.05).

**Table 2:** Table 2. Correlations between alterations in GM in relation to drug dosage, illness duration and symptoms.

Brain regions	RT-SCZ						CT-SCZ		
	Left inferiortemporal tkn	Left middletemporal tkn	Left parahippocampal tkn	Left inferiorparietal vol	Left inferiortemporal vol	Left middletemporal vol	Left frontalpole tkn	Right fusiform tkn	Right inferiortemporal vol
Illness duration	−^a^			−^a^	−^a^	−^a^		−^a^	−^b^
CPZ equivalents (mg)		−^a^							
PANSS total scores		−^a^					−^a^		
PANSS positive scores			−^a^						
PANSS negative scores		−^a^					−^b^		
PANSS general scores		−^a^							

*PANSS* positive and negative syndrome scale, *CPZ* chlorpromazine, *RT-SCZ* risperidone-treated schizophrenia patients, *CT-SCZ* clozapine-treated schizophrenia patients, *tkn* thickness, *vol* volume; −, negative correlation. ^a^*p* < 0.05; ^b^*p* < 0.01.

## Discussion

To our knowledge, this is the first study investigating brain gray matter morphometric alterations between never-treated and long-term monotherapy-treated chronic schizophrenia patients. In general, both treated and never-treated chronic schizophrenia patients, relative to controls, showed cortical GM reductions and increases in similar brain regions, mainly involving bilateral medial and rostral middle frontal, left banks superior temporal sulcus, and left medial occipital cortex regions. Relative to never-treated patients, the two treated chronic patient groups showed widespread reductions mainly in the bilateral prefrontal, temporal, and left inferior parietal lobules. Within the treated group, the clozapine monotherapy group had a more severe reduction mainly in the bilateral prefrontal cortex than the risperidone monotherapy group. In addition, GM reduction was negatively correlated with dosage, duration and symptom severity among treated patients. These findings provide novel insights into the influences of antipsychotic medications on GM abnormalities over the long-term course of illness.

One of the main findings of the current study was the reductions mainly involving bilateral medial and rostral middle frontal, left banks superior temporal sulcus, and left fusiform cortex and increases in the left cuneus in both long-term treated and untreated chronic patients compared to healthy controls. These findings were consistent with previous studies of first-episode schizophrenia before and after short-term treatment^4,6,33,34^, which showed decreases predominantly in the middle and inferior frontal, superior and middle temporal, and anterior cingulate cortex in both treated and untreated patients. This suggests that specific brain regions involved in schizophrenia may be affected in the same way by short- and long-term antipsychotic treatment. In addition, we also found increased GM in the left cuneus in both never-treated and treated chronic patients, which agreed with the previous study of cortical thickness increases in antipsychotic-naïve, first-episode schizophrenia^9^. This might represent a compensatory response related to increased activation in the cuneus cortex to support visual processing in patients with schizophrenia^35,36^. Taken together, the reductions in the bilateral superior temporal lobe and rostral middle frontal lobe cortex and increase in the left cuneus cortex were found both in untreated and treated chronic and first-episode schizophrenia patients. Therefore, these common alterations in brain regions both in chronic and first-episode schizophrenia patients may be relatively stable throughout the course of illness.

By comparing the never-treated with treated chronic schizophrenia patients, it was remarkable that although the whole brain was examined, the reductions were mainly in the bilateral prefrontal and temporal cortex, and left inferior parietal lobule in the treated patients. These findings were partly consistent with previous brain structural studies of short-term treatment in schizophrenia patients^7,34,37,38^, i.e., mainly reductions in the prefrontal and temporal lobes. Furthermore, the prefrontal and temporal cortices have been reported to be associated with the treatment dosage during short-term treatment^7,34,37,38^, are within the dopamine pathway^4^ and show dopaminergic dysregulation^39–41^ in schizophrenia. Therefore, our study further demonstrates that the prefrontal and temporal cortices may be not only short-term treatment targets but also targets for long-term treatment.

Another interesting finding was that the inferior parietal lobule decreased in chronic patients after long-term treatment, which has rarely been reported in previous studies with short-term treatment^18,19,42^. In contrast, some schizophrenia studies with long-term treatment^21,43–45^ have also shown parietal lobe decreases in treated patients^21,45^ and in animal models^43,44^. It is possible that there are differences between short- and long-term antipsychotic treatment effects on the inferior parietal lobule. Therefore, the parietal lobe might be the key target for long-term antipsychotic treatment, which is considered the key area regarding several cognitive and motor functions and tasks involving the integration of different stimuli^46,47^. Furthermore, the inferior parietal lobule has been shown to be involved in various neuropsychological functions affected by schizophrenia and supports the frontal lobe in storing and retrieving verbal information^46^. The reduction in the inferior parietal lobule cortex may be characteristic of schizophrenia patients with long-term treatment, and it could also be a specific target for long-term treatment. Therefore, our findings not only provide evidence for similar brain alterations with short-term and long-term treatment but also the specific alterations of long-term treatment for schizophrenia patients.

It should be noted that unlike our previous findings that long-term untreated patients showed more severe deficits in the white matter^17^ and functional networks^48^ than treated patients, our findings revealed more regions with reduced GM in treated patients than untreated patients. One of the possible explanations is that the mechanism of antipsychotics may be associated with GM reductions and functional performance improvements. Neuroinflammation is one of the pathophysiological mechanisms of schizophrenia^49^, including an increase in microglial activation^50^ and extracellular volume^51^ in GM and white matter. Antipsychotic treatment could promote decreases in activated glia and extracellular volume and improve neuronal function, which has been associated with an anti-inflammatory effect^49,52^. The different principles underlying brain structural and functional MRI may result in different findings. The second possible explanation is that it is mainly gray matter loss that progresses over time, whereas white matter deficits are stable or may even improve over the course of the illness^49^. GM loss progresses further after the onset of schizophrenia^53^, and antipsychotics may not counteract the pathophysiologic processes leading to the GM loss underlying schizophrenia^21^. Taken together, these findings suggest the possibility of different effects of treatment on GM and white matter, which may be the focus of significant future work.

Another contribution of the current study is that the patients with long-term clozapine monotherapy displayed more severe and extensive GM loss than the patients with risperidone monotherapy. These findings were similar to those of a previous study^54^; for example, cortical thinning of the pars triangularis was found in first-episode schizophrenia patients with 2 years of clozapine treatment, and no significant differences were found in patients with risperidone treatment. However, our study suggests that the CT-SCZ group had more severe reductions mainly in the bilateral prefrontal cortex and left cuneus and fewer decreases in the left ventral temporal lobe than the RT-SCZ group. One possible interpretation is that the effects of antipsychotic treatment may be cumulative over the years of the illness course or more pronounced later in the course of the illness^17^. Although it is nearly impossible to have chronically treated patients with monotherapy during the whole illness, we recruited stably treated patients with monotherapy for at least the most recent 5 years, which could represent the long-term treatment effect on the brain. Therefore, our findings may suggest a specific and different effect of long-term risperidone and clozapine treatment on this pathway.

The current study also revealed that GM reductions were negatively correlated with dosage, duration and symptom severity among the treated patients, involving the inferior and middle temporal lobes, inferior parietal lobe, and fusiform cortex regions. This finding suggests that widespread cortical deficits occurring in the later stages of schizophrenia may be crucial to the pathogenesis of the disease. The anatomical deficits in the inferior parietal lobe may represent the core pathology during the later course of schizophrenia. Previous studies have shown that the above cortical regions were associated with symptoms of schizophrenia^11,35,46,55,56^. Therefore, the findings indicate that cumulative antipsychotic exposure, illness duration, and symptom severity may be potentially contributing factors influencing the progression of GM deficits in chronic schizophrenia patients.

Several limitations should be considered in interpreting our findings. First, the GM differences among the three patient groups and their relationships with treatment history are constrained by the lack of random assignment to long-term treatment versus no treatment conditions. However, such a study would not be feasible or ethically justified. Therefore, our cross-sectional approach may be the only feasible strategy for addressing questions about the long-term course of schizophrenia and antipsychotic effects on brain structure. Second, we note that the sex ratio is significantly different among the 4 groups. Previous studies^57–59^ explored the impact of sex differences on the brain features of schizophrenia, but the results are inconsistent, even a study^58^ reported no significant differential sex effects in schizophrenia for either GM cortical thickness or subcortical volume development. In this study, we took age and sex as covariates by ANCOVA analysis to control for potential confounding effects and found that sex has no interactive effect on the results. Therefore, the results of the differences between groups may result from long-term antipsychotic treatment, rather than sex differences. Although all patients were well matched on variables such as age, duration, age at onset, and dosage, these issues need to be considered when interpreting our findings. Third, there were no significant differences in subcortical volumes after multiple comparisons. Subcortical structures may not have been accurately segmented^60^ and may not significantly change as a whole. Therefore, it is necessary to adjust the segmentation and perform further subarea analysis. Lastly, to clarify mechanisms of these effects, animal model research will be needed to examine longitudinal long-term differential antipsychotic effects on GM to experimentally confirm a relationship between antipsychotics and GM.

In conclusion, our findings provide new insights into the understanding of the long-term treatment of chronic schizophrenia and the different brain structural manifestations after different medications. These findings of long-term treatment were similar to those in short-term studies in treated schizophrenia, mainly involving the temporal lobe and prefrontal lobe. Both treated and never-treated chronic schizophrenia patients showed cortical gray matter reductions and increases in similar brain regions, and the reductions in the inferior parietal lobule may be characteristic of schizophrenia patients with long-term treatment. Our study also suggests that patients with long-term clozapine monotherapy displayed more severe and extensive GM loss in the bilateral prefrontal and left cuneus cortex than patients with risperidone monotherapy. These findings deepen our understanding of the effects of long-term treatment and may provide evidence for the development of new medications or potential treatment targets for long-term treatment in schizophrenia patients.

## Supplementary information

Supplementary table S1

## References

[1] Lui S, Zhou XJ, Sweeney JA, Gong Q (2016). Psychoradiology: the frontier of neuroimaging in psychiatry. Radiology.

[2] Lui S (2009). Association of cerebral deficits with clinical symptoms in antipsychotic-naive first-episode schizophrenia: an optimized voxel-based morphometry and resting state functional connectivity study. Am. J. Psychiatry.

[3] Lui S (2010). Short-term effects of antipsychotic treatment on cerebral function in drug-naive first-episode schizophrenia revealed by “resting state” functional magnetic resonance imaging. Arch. Gen. Psychiatry.

[4] Gong Q, Lui S, Sweeney JA (2016). A selective review of cerebral abnormalities in patients with first-episode schizophrenia before and after treatment. Am. J. Psychiatry.

[5] Millan MJ (2016). Altering the course of schizophrenia: progress and perspectives. Nat. Rev. Drug Discov..

[6] Dietsche B, Kircher T, Falkenberg I (2017). Structural brain changes in schizophrenia at different stages of the illness: a selective review of longitudinal magnetic resonance imaging studies. Aust. N. Z. J. Psychiatry.

[7] Fusar-Poli P (2013). Progressive brain changes in schizophrenia related to antipsychotic treatment? A meta-analysis of longitudinal MRI studies. Neurosci. Biobehav. Rev..

[8] Vita A, De Peri L, Deste G, Barlati S, Sacchetti E (2015). The effect of antipsychotic treatment on cortical gray matter changes in schizophrenia: does the class matter? A meta-analysis and meta-regression of longitudinal magnetic resonance imaging studies. Biol. Psychiatry.

[9] Xiao Y (2015). Altered cortical thickness related to clinical severity but not the untreated disease duration in schizophrenia. Schizophr. Bull..

[10] Pantelis C (2003). Neuroanatomical abnormalities before and after onset of psychosis: a cross-sectional and longitudinal MRI comparison. Lancet.

[11] Onitsuka T (2004). Middle and inferior temporal gyrus gray matter volume abnormalities in chronic schizophrenia: an MRI study. Am. J. Psychiatry.

[12] Ellison-Wright I, Glahn DC, Laird AR, Thelen SM, Bullmore E (2008). The anatomy of first-episode and chronic schizophrenia: an anatomical likelihood estimation meta-analysis. Am. J. Psychiatry.

[13] Chan RC, Di X, McAlonan GM, Gong QY (2011). Brain anatomical abnormalities in high-risk individuals, first-episode, and chronic schizophrenia: an activation likelihood estimation meta-analysis of illness progression. Schizophr. Bull..

[14] van Haren NE (2011). Changes in cortical thickness during the course of illness in schizophrenia. Arch. Gen. Psychiatry.

[15] Torres US (2016). Patterns of regional gray matter loss at different stages of schizophrenia: a multisite, cross-sectional VBM study in first-episode and chronic illness. Neuroimage Clin..

[16] Zhang W (2015). Brain structural abnormalities in a group of never-medicated patients with long-term schizophrenia. Am. J. Psychiatry.

[17] Xiao Y (2018). White matter abnormalities in never-treated patients with long-term schizophrenia. Am. J. Psychiatry.

[18] Lieberman JA (2005). Antipsychotic drug effects on brain morphology in first-episode psychosis. Arch. Gen. Psychiatry.

[19] Moncrieff J, Leo J (2010). A systematic review of the effects of antipsychotic drugs on brain volume. Psychol. Med..

[20] Arango C (2012). Progressive brain changes in children and adolescents with first-episode psychosis. Arch. Gen. Psychiatry.

[21] Ho BC, Andreasen NC, Ziebell S, Pierson R, Magnotta V (2011). Long-term antipsychotic treatment and brain volumes: a longitudinal study of first-episode schizophrenia. Arch. Gen. Psychiatry.

[22] Veijola J (2014). Longitudinal changes in total brain volume in schizophrenia: relation to symptom severity, cognition and antipsychotic medication. PLoS ONE.

[23] Singh SP (2005). Determining the chronology and components of psychosis onset: The Nottingham Onset Schedule (NOS). Schizophr. Res..

[24] Bowie CR, Grossman M, Gupta M, Oyewumi LK, Harvey PD (2014). Cognitive remediation in schizophrenia: efficacy and effectiveness in patients with early versus long-term course of illness. Early Inter. Psychiatry.

[25] Deste G (2019). Effectiveness of cognitive remediation in early versus chronic schizophrenia: a preliminary report. Front. Psychiatry.

[26] Kay SR, Fiszbein A, Opler LA (1987). The positive and negative syndrome scale (PANSS) for schizophrenia. Schizophr. Bull..

[27] Gardner DM, Murphy AL, O’Donnell H, Centorrino F, Baldessarini RJ (2010). International consensus study of antipsychotic dosing. Am. J. Psychiatry.

[28] Dale AM, Fischl B, Sereno MI (1999). Cortical surface-based analysis. I. Segmentation and surface reconstruction. Neuroimage.

[29] Fischl B, Liu A, Dale AM (2001). Automated manifold surgery: constructing geometrically accurate and topologically correct models of the human cerebral cortex. IEEE Trans. Med. Imaging.

[30] Ségonne F, Pacheco J, Fischl B (2007). Geometrically accurate topology-correction of cortical surfaces using nonseparating loops. IEEE Trans. Med. Imaging.

[31] Reuter M, Schmansky NJ, Rosas HD, Fischl B (2012). Within-subject template estimation for unbiased longitudinal image analysis. Neuroimage.

[32] Desikan RS (2006). An automated labeling system for subdividing the human cerebral cortex on MRI scans into gyral based regions of interest. Neuroimage.

[33] Mané A (2009). Progressive gray matter changes in first episode schizophrenia: a 4-year longitudinal magnetic resonance study using VBM. Schizophr. Res..

[34] Lesh TA (2015). A multimodal analysis of antipsychotic effects on brain structure and function in first-episode schizophrenia. JAMA Psychiatry.

[35] Goghari VM, Rehm K, Carter CS, MacDonald AW (2007). Regionally specific cortical thinning and gray matter abnormalities in the healthy relatives of schizophrenia patients. Cereb. Cortex..

[36] Van Snellenberg JX (2016). Mechanisms of working memory impairment in schizophrenia. Biol. Psychiatry.

[37] Leung M (2011). Gray matter in first-episode schizophrenia before and after antipsychotic drug treatment. Anatomical likelihood estimation meta-analyses with sample size weighting. Schizophr. Bull..

[38] Shah C (2017). Common pattern of gray-matter abnormalities in drug-naive and medicated first-episode schizophrenia: a multimodal meta-analysis. Psychol. Med..

[39] Goldsmith SK, Shapiro RM, Joyce JN (1997). Disrupted pattern of D2 dopamine receptors in the temporal lobe in schizophrenia. A postmortem study. Arch. Gen. Psychiatry.

[40] Laviolette SR (2007). Dopamine modulation of emotional processing in cortical and subcortical neural circuits: evidence for a final common pathway in schizophrenia. Schizophr. Bull..

[41] Glantz LA (2010). Pro-apoptotic Par-4 and dopamine D2 receptor in temporal cortex in schizophrenia, bipolar disorder and major depression. Schizophr. Res..

[42] Molina V (2005). Increase in gray matter and decrease in white matter volumes in the cortex during treatment with atypical neuroleptics in schizophrenia. Schizophr. Res..

[43] Dorph-Petersen KA (2005). The influence of chronic exposure to antipsychotic medications on brain size before and after tissue fixation: a comparison of haloperidol and olanzapine in macaque monkeys. Neuropsychopharmacology.

[44] Konopaske GT (2007). Effect of chronic exposure to antipsychotic medication on cell numbers in the parietal cortex of macaque monkeys. Neuropsychopharmacology.

[45] Huhtaniska S. et al. Long-term antipsychotic use and brain changes in schizophrenia - a systematic review and meta-analysis. *Hum. Psychopharmacol*. 10.1002/hup.2574 (2017). 10.1002/hup.2574 28370309

[46] Yildiz M, Borgwardt SJ, Berger GE (2011). Parietal lobes in schizophrenia: do they matter. Schizophr. Res. Treat..

[47] Teixeira S (2014). Integrative parietal cortex processes: neurological and psychiatric aspects. J. Neurol. Sci..

[48] Yao L (2019). Functional brain networks in never-treated and treated long-term Ill schizophrenia patients. Neuropsychopharmacology.

[49] Kahn RS, Sommer IE (2015). The neurobiology and treatment of first-episode schizophrenia. Mol. Psychiatry.

[50] van Berckel BN (2008). Microglia activation in recent-onset schizophrenia: a quantitative (R)-[11C]PK11195 positron emission tomography study. Biol. Psychiatry.

[51] Pasternak O (2012). Excessive extracellular volume reveals a neurodegenerative pattern in schizophrenia onset. J. Neurosci..

[52] Tourjman V (2013). Antipsychotics’ effects on blood levels of cytokines in schizophrenia: a meta-analysis. Schizophr. Res..

[53] Hulshoff Pol HE, Kahn RS (2008). What happens after the first episode? A review of progressive brain changes in chronically ill patients with schizophrenia. Schizophr. Bull..

[54] Molina V, Taboada D, Aragüés M, Hernández JA, Sanz-Fuentenebro J (2014). Greater clinical and cognitive improvement with clozapine and risperidone associated with a thinner cortex at baseline in first-episode schizophrenia. Schizophr. Res..

[55] Shenton ME, Dickey CC, Frumin M, McCarley RW (2001). A review of MRI findings in schizophrenia. Schizophr. Res..

[56] Lee CU (2002). Fusiform gyrus volume reduction in first-episode schizophrenia: a magnetic resonance imaging study. Arch. Gen. Psychiatry.

[57] Kaczkurkin AN, Raznahan A, Satterthwaite TD (2019). Sex differences in the developing brain: insights from multimodal neuroimaging. Neuropsychopharmacology.

[58] Weisinger B (2013). Lack of gender influence on cortical and subcortical gray matter development in childhood-onset schizophrenia. Schizophr. Bull..

[59] Lang XE (2018). Sex difference in association of symptoms and white matter deficits in first-episode and drug-naive schizophrenia. Transl. Psychiatry.

[60] Babalola KO (2009). An evaluation of four automatic methods of segmenting the subcortical structures in the brain. Neuroimage.

