# Supplementary material for: Characteristics of gray matter alterations in never-treated and treated chronic schizophrenia patients
Source: Transl Psychiatry. 2020 May 12;10:136. doi: 10.1038/s41398-020-0828-4 (PMC7217843; doi:10.1038/s41398-020-0828-4)
Supplement: Supplementary file 1 — Supplementary table S1 [file 41398_2020_828_MOESM1_ESM.doc]

**Supplementary Table S1. GM in cortical thickness, surface area and volume showing differences among never-treated long-term schizophrenia patients, risperidone-treated patients, clozapine-treated patients, and healthy controls**

|  | RT-SCZ vs. NT-SCZ | | | | | | CT-SCZ vs. NT-SCZ | | | | | | RT-SCZ vs. CT-SCZ | | | | | | NT-SCZ vs. HC | | | | | | RT-SCZ vs. HC | | | | | | CT-SCZ vs. HC | | | | | |
| --- | --- | --- | --- | --- | --- | --- | --- | --- | --- | --- | --- | --- | --- | --- | --- | --- | --- | --- | --- | --- | --- | --- | --- | --- | --- | --- | --- | --- | --- | --- | --- | --- | --- | --- | --- | --- |
| Brain region | L tkn | R tkn | L area | R area | L vol | R vol | L tkn | R tkn | L area | R area | L vol | R vol | L tkn | R tkn | L area | R area | L vol | R vol | L tkn | R tkn | L area | R area | L vol | R vol | L tkn | R tkn | L area | R area | L vol | R vol | L tkn | R tkn | L area | R area | L vol | R vol |
| banks sts |  |  |  |  |  |  | - |  |  |  |  |  |  |  |  |  |  |  |  |  | - |  | - |  |  | - | - |  | - |  | - | - | - |  | - |  |
| caudal anterior cingulate |  |  |  |  |  |  |  |  |  |  |  |  |  |  |  |  |  |  |  |  |  |  |  |  |  |  |  |  |  |  |  |  |  |  |  |  |
| caudal middle frontal |  |  |  |  |  |  |  |  |  |  |  |  |  |  |  |  |  |  |  |  |  |  |  |  |  |  |  |  |  |  |  |  |  |  |  |  |
| cuneus |  |  |  |  |  |  |  |  |  |  |  |  | + |  |  |  |  |  | + |  |  |  |  |  |  |  | + |  |  |  |  |  |  |  |  |  |
| entorhinal |  | - |  |  | - |  |  | - |  |  |  |  | - |  |  |  | - |  |  |  |  |  |  |  |  | - |  |  | - |  |  | - |  |  |  |  |
| fusiform |  | - |  |  | - |  |  | - |  |  |  |  |  |  |  |  |  |  |  |  | - |  |  |  |  |  | - |  |  | - |  | - |  |  |  | - |
| inferior parietal |  |  |  |  | - |  | - |  |  |  |  |  |  |  |  |  |  |  |  |  |  |  |  |  |  |  |  |  | - | - | - | - |  |  | - | - |
| inferior temporal | - | - | - |  | - | - | - | - | - |  | - | - |  |  |  |  |  |  |  |  |  |  |  |  |  |  | - |  | - | - | - | - | - |  | - | - |
| isthmus cingulate |  |  |  |  |  |  |  |  |  |  |  |  |  |  |  |  |  |  |  |  |  |  |  |  |  |  |  |  |  |  |  |  |  |  |  |  |
| lateral occipital |  |  |  |  |  |  |  |  |  |  |  |  |  |  |  |  |  |  |  |  |  |  |  |  |  |  |  |  |  |  |  |  |  |  |  |  |
| lateral orbitofrontal | - |  |  |  |  |  | - | - |  |  | - |  | + | + |  |  |  |  |  |  |  |  |  |  | - |  |  |  | - |  | - | - |  |  | - |  |
| lingual |  |  | - |  |  |  |  |  |  |  |  | - |  |  |  |  |  |  |  |  |  |  |  |  |  |  | - |  | - | - |  |  |  |  | - | - |
| medial orbitofrontal |  |  |  |  |  |  | - | - |  |  |  | - | + | + |  |  |  | + |  |  | - |  | - |  |  |  | - |  | - |  | - | - | - |  | - | - |
| middle temporal | - | - |  |  | - |  | - | - |  |  | - |  |  |  |  |  |  |  |  |  |  |  |  |  | - | - |  |  | - | - | - | - |  |  | - | - |
| parahippocampal | - | - |  |  |  |  |  | - |  |  |  |  | - |  |  |  |  |  |  |  |  |  |  |  | - | - |  |  |  |  |  | - |  |  |  |  |
| paracentral |  |  |  |  |  |  |  |  |  |  |  |  |  |  |  |  |  |  |  |  |  |  |  |  |  |  |  |  |  |  |  |  |  |  |  |  |
| pars opercularis |  |  |  |  |  |  | - | - |  |  |  |  | + | + |  |  |  |  |  |  |  |  |  |  |  |  |  |  |  |  | - | - |  |  |  |  |
| pars orbitalis |  | - |  |  |  |  |  | - |  |  |  | - |  |  |  |  |  |  |  |  |  |  |  |  | - | - |  |  |  |  | - | - |  |  |  | - |
| pars triangularis |  |  |  |  |  |  | - | - |  |  |  |  | + |  |  |  |  |  |  |  |  |  |  |  | - |  |  |  |  |  | - | - |  |  | - |  |
| pericalcarine |  |  |  |  |  |  |  |  |  |  |  |  |  |  |  |  |  |  |  |  | - |  |  |  |  |  |  |  |  |  |  |  | - |  |  |  |
| postcentral |  |  |  |  |  |  |  |  |  |  |  |  |  |  |  |  |  |  |  |  |  |  |  |  |  |  |  |  |  |  |  |  |  |  |  |  |
| posterior cingulate |  |  |  |  |  |  |  |  |  |  |  |  |  |  |  |  |  |  |  |  |  |  |  |  |  |  |  |  |  |  |  |  |  |  |  |  |
| precentral |  |  |  |  |  |  |  |  |  |  |  |  |  |  |  |  |  |  |  |  |  |  |  |  |  |  |  |  |  |  |  |  |  |  |  |  |
| precuneus |  |  |  |  |  |  |  |  |  |  |  |  |  |  |  |  |  |  |  |  |  |  |  |  |  |  |  |  |  |  |  |  |  |  |  |  |
| rostral anterior cingulate |  |  |  |  |  |  |  |  |  |  |  |  |  |  |  |  |  |  |  |  |  | - |  | - |  |  |  | - |  | - |  |  |  |  |  | - |
| rostral middle frontal |  |  |  |  |  |  | - | - |  |  |  |  | + | + |  |  |  |  | - | - |  |  |  |  | - | - |  |  |  |  | - | - |  |  | - |  |
| superior frontal | - |  |  |  |  |  | - | - |  |  | - |  |  | + |  |  |  |  |  |  |  |  |  |  | - | - |  |  | - |  | - | - |  |  | - |  |
| superior parietal |  |  |  |  |  |  |  |  |  |  |  |  |  |  |  |  |  |  |  |  |  |  |  |  |  |  |  |  |  |  |  |  |  |  |  |  |
| superior temporal |  |  |  |  |  |  |  |  |  |  | - |  |  |  |  |  |  |  |  |  |  |  |  |  |  |  |  |  |  |  |  |  | - |  | - |  |
| supramarginal |  |  |  |  |  |  |  |  | - |  | - |  |  |  |  |  |  |  |  |  |  |  |  |  |  |  | - |  | - |  |  |  | - |  | - |  |
| frontal pole |  | - |  |  |  |  | - | - |  |  | - | - |  |  |  |  | + |  |  |  |  |  | + | + |  | - |  |  |  |  | - | - |  |  | - | - |
| temporal pole |  |  |  |  |  |  |  |  |  |  |  |  |  |  |  |  |  |  |  |  |  |  |  |  |  |  |  |  |  |  |  |  |  |  |  |  |
| transverse temporal |  |  |  |  | - |  |  |  | - |  | - |  |  |  |  |  |  |  |  |  |  |  |  |  |  |  | - |  | - |  |  |  | - |  | - |  |
| insula |  |  |  |  |  |  |  |  |  |  |  |  |  |  |  |  |  |  |  |  |  |  |  |  |  |  |  |  |  |  |  |  |  |  |  |  |

Analysis of covariance (ANCOVA) was conducted using a general linear model followed by least significant difference post-hoc pairwise comparisons after controlling for age and gender. The statistical significance level was set at p = 0.05, controlled for multiple comparisons using a false discovery rate criterion.

Abbreviations: RT-SCZ, risperidone-treated schizophrenia patients; CT-SCZ, clozapine-treated schizophrenia patients; HC, healthy controls; NT-SCZ, never treated schizophrenia patients; L, left hemisphere; R, right hemisphere; sts: superior temporal sulcus; tkn: thickness; vol, volume; -, decrease ; +, increase.
